# Is the High Frequency of Machado-Joseph Disease in China Due to New Mutational Origins?

**DOI:** 10.3389/fgene.2018.00740

**Published:** 2019-02-20

**Authors:** Tianjiao Li, Sandra Martins, Yun Peng, Puzhi Wang, Xiaocan Hou, Zhao Chen, Chunrong Wang, Zhaoli Tang, Rong Qiu, Chao Chen, Zhengmao Hu, Kun Xia, Beisha Tang, Jorge Sequeiros, Hong Jiang

**Affiliations:** ^1^Department of Neurology, Xiangya Hospital, Central South University, Changsha, China; ^2^IPATIMUP – Institute of Molecular Pathology and Immunology of the University of Porto, Instituto de Investigação e Inovação em Saúde (i3S), Porto, Portugal; ^3^School of Information Science and Engineering, Central South University, Changsha, China; ^4^Laboratory of Medical Genetics, Central South University, Changsha, China; ^5^National Clinical Research Center for Geriatric Diseases, Xiangya Hospital, Central South University, Changsha, China; ^6^Key Laboratory of Hunan Province in Neurodegenerative Disorders, Central South University, Changsha, China; ^7^Parkinson’s Disease Center of Beijing Institute for Brain Disorders, Beijing, China; ^8^Collaborative Innovation Center for Brain Science, Shanghai, China; ^9^Collaborative Innovation Center for Genetics and Development, Shanghai, China; ^10^IBMC – Institute for Molecular and Cell Biology, i3S – Instituto de Investigação e Inovação em Saúde, ICBAS (Instituto de Ciências Biomédicas Abel Salazar), University of Porto, Porto, Portugal

**Keywords:** spinocerebellar ataxia type 3, SCA3, Machado-Joseph disease, founder effect, haplotype, mutational origins

## Abstract

Machado-Joseph disease (MJD, also known as spinocerebellar ataxia 3 or SCA3) is the most common dominant ataxia worldwide, with an overall average prevalence of 1–5/100,000. To this date, two major ancestral lineages have been found throughout the world. In China, the relative frequency of MJD among the SCAs reaches as high as 63%, however, little is known about its mutational origin in this country. We analyzed 50 families with MJD patients in two or more generations to study the hypothesis that new mutational events have occurred in this population. Haplotypes based on 20 SNPs have shown new genetic backgrounds segregating with MJD mutations in our cohort from China. We found the “Joseph-derived” lineage (Joseph lineage with a G variant in rs56268847) to be very common among Chinese MJD patients. Moreover, we estimated the time for the origin of this MJD SNP background based on STR diversity flanking the (CAG)_n_ of *ATXN3*. It was surprising to find that the Chinese MJD population originated from 8,000 to 17,000 years ago, far earlier than the previous literature reports, which will be an important evidence to explain the origin, spread and founder effects of MJD.

## Introduction

Machado-Joseph disease (MJD, OMIM#109150), also known as spinocerebellar ataxia type 3 (SCA3), is one of the polyQ diseases. It is a rare autosomal dominantly inherited neurodegenerative disease that causes progressive cerebellar ataxia, resulting in lacks of muscle control and coordination of the upper and lower extremities, with symptoms including dysarthria, dysphagia, pyramidal signs, progressive external ophthalmoplegia, and dystonia (Bettencourt and Lima, 2011; Costa Mdo and Paulson, 2012; Wang et al., 2015; Paulson et al., 2017). This autosomal dominant disease is caused by an elongated polyglutamine stretch encoded by more than 51 CAG repeats on the *ATXN3* allele (Rüb et al., 2013; Ding et al., 2016; Chen et al., 2017; Long et al., 2018). MJD is spread worldwide, but it has a higher relative frequency among the SCAs in Portugal (57.8%) (Vale et al., 2010), Brazil (59.6%) (de Castilhos et al., 2014), Japan (43%) (Takano et al., 1998), and Germany (42%) (Schöls et al., 1997). Our previous study has shown that mainland China has a high prevalence of MJD with a relative frequency of 62.6% (Chen et al., 2018).

This disease was first reported in an extended family of Portuguese-Azorean ancestry, in 1972 (Nakano et al., 1972), and was believed to be very frequent in Portugal due to founder effects. Also, Japan, Brazil and France all had founder effects of MJD (Marie-Françoise Chesselet, 2001). Furthermore, a linkage disequilibrium analysis was carried out with three single nucleotide polymorphisms (SNPs, A^669^TG/G^669^TG, C^987^GG/G^987^GG, and TAA^1118^/TAC^1118^) and five short tandem repeats (STRs, D14S1015, D14S995, D14S973, D14S1016, and D14S977), in 249 families of various ethnic backgrounds. Four different SNP haplotypes were identified segregating with MJD expansions: A-C-A, A-G-A, G-G-A, and G-G-C (Gaspar et al., 2001). Following this discovery, a worldwide study of extended haplotypes was performed in 264 MJD families, and two major ancestral lineages were confirmed: the GTGGCA background or the Machado lineage, probably originated in Portugal; and the TTACAC or Joseph lineage, observed in 19 countries, including Japan (Martins et al., 2007). To determine the occurrence of new mutation events and clarify the spread of ancestral MJD lineages in different populations, genetic distances were determined using a total of 20 SNPs and 4 microsatellites for the purpose of haplotypes identification (Martins et al., 2012), however, little information on MJD haplotypes is available in China, although the frequency of MJD is very high in this Asian population.

In this study, to determine the origin and estimate the age of MJD mutational event(s) in China, we performed a haplotype analysis of 20 SNPs and 7 microsatellites in 50 Chinese MJD families.

## Materials and Methods

### Patients and Controls

We analyzed 50 families with MJD, from Southern China, including 109 patients, and 105 healthy individuals. All participants agreed with our request for collection of peripheral blood samples and signed an informed consent form. The study was approved by Ethics Committee of Xiangya Hospital of Central South University in China. Genomic DNA was isolated using a standard protocol. Each family has at least two generations and has at least four individuals. Healthy spouses served as the control group. In one of the families studied, a patient homozygous for an MJD expansion was analyzed together with both parents and his sister.

### Genotyping and Haplotype Analysis

**FIGURE 1:**
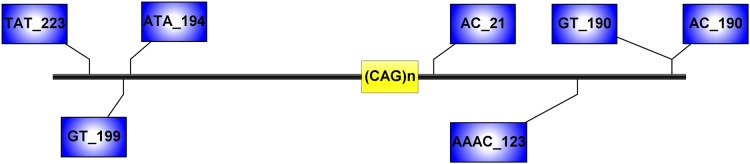
Microsatellite loci flanking the (CAG)n of *ATXN3*, analyzed in this study. Distances from the (CAG)n, included in the STR name, are expressed in kilobases.

We analyzed 20 SNPs located upstream and downstream the (CAG)n, including the previously analyzed six core SNPs and variants within a 4 kb area flanking the repeat (Figure 1; Martins et al., 2006; Costa et al., 2019). PCR amplification reactions were done with TSE 101 Golden Star T6 Super PCR Mix (1.1×). The location of all loci and Primers for amplification are listed in the Supplementary Table S1. Genotyping of SNPs was performed through Sanger sequencing. We inferred allelic phases by segregation for most of analyzed families; some haplotypes were reconstructed by PHASE software version 2.1.1^1^ (only haplotypes with probabilities greater than 0.6 were used for subsequent analyses). We compared the distribution of SNPs in the Chinese MJD population with the two ancestral lineages described previously, and selected four different SNPs among these to test for Hardy-Weinberg equilibrium and Chi-square test (X^2^). In this test, 49 healthy spouses of probands served as controls. We first distinguished the haplotypes of the six core SNPs and conducted a Fisher’s exact test. Basically, the formula δ = (Fd-Fc)/ (1-Fc) is used to calculate the approximate risk of population attribution and provide evidence of LD. Then, we extended the haplotype to 20 SNPs for further analysis (Devlin and Risch, 1995; Gaspar et al., 2001).

### STR-Based Haplotype Analysis and Age Estimation

Capillary electrophoresis was used to detect the seven STRs (TAT_223)n, (GT_199)n, (ATA_194)n, (AC_21)n, (AAAC)n, (GT)n, and (AC_190)n (Figure 1), and haplotypes reconstructed by segregation and with the PHASE software version 2.1.1 (only haplotypes with probabilities greater than 0.6 were used for subsequent analyses). We determine ancestral haplotypes and steps of mutation by STR-based haplotype from the most common haplotype based on 20 SNPs and assess the age of mutation events. This formula 𝜀 = 1-[(1-c) (1-μ)] is used to calculate the probability of change for each generation, where c represents the recombination rate and μ represents the mutation rate. The multiplication of 𝜀 and t yields the average of mutations and reorganizations (λ), where t is the number of generations (Martins et al., 2007).

## Results

### SNP Haplotyping of Chinese MJD Families

We identified 13 disease-associated haplotypes in our cohort of Chinese MJD families. We compared these haplotypes with the Joseph lineage and indicated their differences (Table 1). It is worthy of mentioning that, for four SNPs, we found new alleles segregating with expanded alleles, not previously associated to Machado or Joseph ancestral MJD lineages (Table 2). The G allele occurs in the rs56268847 of the pathogenic chromosomes in the 28 families, exceeding 50% of the total fifty families involved in the study. Regarding SNPs rs16999141, rs10467857 and rs77086230, alleles never found in phase with expanded MJD alleles were here observed in families 21, 31 and 15, respectively.

**Table 1:** SNP-based haplotypes of MJD Chinese families in comparison to Joseph MJD lineage.

SNP	refSNP ID	Joseph lineage	Haplotypes of MJD Chinese families^abc^												
			
			A (*n* = 10)	B (*n* = 12)	C (*n* = 1)	D (*n* = 8)	E (*n* = 1)	F (*n* = 1)	G (*n* = 11)	H (*n* = 1)	I (*n* = 1)	J (*n* = 1)	K (*n* = 2)	L (*n* = 1)	M (*n* = 1)
IVS6-30G > T	rs12590497	T	T	T	T	T	T	T	T	T	T	**G**	**G**	**G**	T
GTT^527^/GTC^527^	rs16999141	T	T	T	T	T	T	T	T	T	T	T	T	**C**	T
IVS8-86T > G	rs10146519	G	G	G	G	G	G	G	G	G	G	G	G	G	G
A^669^TG/G^669^TG	rs1048755	A	A	A	A	A	A	A	A	**G**	A	A	A	A	**G**
C/T.124	rs12586535	T	T	T	T	T	T	**C**	T	T	T	T	T	T	T
T/C.248	rs12586471	C	C	C	C	C	C	**T**	C	C	C	C	C	C	C
A/G.485	rs56268847	A	A	**G**	**G**	A	A	A	**G**	**G**	**G**	**G**	A	A	**G**
G/A.868	rs10467858	A	A	A	A	A	A	A	A	A	A	A	A	A	**G**
C/G.910	rs10467857	G	G	G	G	G	**C**	G	G	G	G	G	G	G	G
T/C.921	rs10467856	C	C	C	C	C	C	C	C	C	C	C	C	C	C
**(CAG)_n_**
C^987^GG/G^987^GG	rs12895357	C	C	C	C	**G**	**G**	**G**	**G**	**G**	**G**	**G**	**G**	**G**	C
TAA^1118^/TAC^1118^	rs7158733	A	A	A	A	A	A	A	A	A	**C**	A	A	A	A
C^1178^/A^1178^	rs3092822	C	C	C	C	C	C	C	C	C	C	C	C	C	C
C/T	rs77086230	C	C	C	**T**	C	C	C	C	C	C	C	C	C	C
C/T	rs79316375	C	C	C	C	C	C	C	C	C	C	C	C	C	C
G/A.3738	rs8004149	A	A	A	A	A	A	A	A	A	A	A	A	A	A
G/A.3770	rs111735934	G	G	G	G	G	G	G	G	G	G	G	G	G	G
C/T.3874	rs181752420	C	C	C	C	C	C	C	C	C	C	C	C	C	C
A/G.3912	rs7142326	G	G	G	G	G	G	G	G	G	G	G	G	G	G
C/T.3980	rs74071847	C	C	C	C	C	C	C	C	C	C	C	C	C	C


*SNP, single-nucleotide polymorphism.*

*^a^A to M indicate different haplotypes observed in Chinese MJD patients. n is the number of families. Haplotype A: families 1, 5, 8, 10, 11, 24, 29, 34, 44, 47; Haplotype B: families 4, 16, 23, 25, 30, 35, 37, 40, 41, 45, 48, 49; Haplotype C: family 15; Haplotype D: families 1, 3, 7, 9#, 19, 26, 38#, 50; Haplotype E: family 31; Haplotype F: family 14; Haplotype G: families 6, 13, 17, 22, 27, 32, 33#, 36, 42, 43, 46; Haplotype H: family 18; Haplotype I: family 12; Haplotype J: family 2; Haplotype K: families 20, 39#; Haplotype L: family 21; Haplotype M: family 28# (# indicate families with some alleles inferred by PHASE).*

*^b^In family 1, both parents are affected and the patient harbours two different MJD lineages.*

*^c^Bold indicates different SNP alleles when compared to the Joseph lineage*.

**Table 2:** Allele frequency of 4 different SNPs analyzed by Hardy-Weinberg equilibrium and chi-square test.

refSNP ID	Group	N^a^		Genotype^b^		*P*-value^c^	Allele^d^		*P*-value^e^
**rs16999141**			**TT**	**TC**	**CC**		**T**	**C**	
	MJD	109	57	52	0	0.005	166 (0.76)	52 (0.24)	<0.001
	Control^f^	49	12	29	8	0.407	53 (0.54)	45 (0.46)	
**rs56268847**			**AA**	**AG**	**GG**		**A**	**G**	
	MJD	109	46	60	3	0.007	152 (0.70)	66 (0.30)	<0.001
	Control^f^	49	43	5	1	0.271	91 (0.93)	7 (0.07)	
**rs10467857**			**GG**	**GC**	**CC**		**G**	**C**	
	MJD	109	56	53	0	0.004	165 (0.76)	53 (0.24)	<0.001
	Control^f^	49	13	26	10	0.901	52 (0.53)	46 (0.47)	
**rs77086230**			**CC**	**CT**	**TT**		**C**	**T**	
	MJD	109	103	6	0	0.957	212 (0.97)	6 (0.03)	<0.001
	Control^f^	49	44	5	0	0.932	93 (0.95)	5 (0.05)	


*MJD, Machado-Joseph disease; SNP, single-nucleotide polymorphism.*

*^a^The number of each group.*

*^b^Value of the Hardy-Weinberg equilibrium for each comparison (MJD vs. controls).*

*^c^The P-value of Hardy-Weinberg equilibrium.*

*^d^Value of the Hardy-Weinberg equilibrium for each comparison (MJD vs. controls).*

*^e^The P-value of chi-square test.*

*^f^49 healthy spouses of probands of 50 Chinese families with MJD served as controls.*

### Hardy-Weinberg Equilibrium and Chi-Square Test for the 4 of SNPs

Hardy-Weinberg equilibrium and chi-square test were carried out to examine the SNP variations. The control group consisting of 49 spouses confirmed by the Hardy-Weinberg equilibrium that satisfy the genetic equilibrium in the four heterogeneous SNPs. In rs16999141, no genotype CC was found in MJD patients, 57 of 109 patients shared TT, and the rest were TC. For SNP rs56268847, significant variation from AA, AG to GG was detected with the *p*-value of the Hardy-Weinberg equilibrium at this position is 0.007. More specifically, 46 out of 109 SCA3/MJD patients had a genotype of AA, while 60 patients had AG, and by contrast, only three patients had GG. Different distribution for SNP rs10467857 was detected. Of the 109 identical patients, 56 are genotyped as GG and 53 as GC (*p*-value of the Hardy-Weinberg equilibrium 0.004), respectively. Similar situation was found in the SNP rs77086230 in a way that Only CC and CT were discovered with a predominance of CC over CT. The *p*-value of Hardy-Weinberg equilibrium of this SNP in SCA3/MJD group and three SNPs in the control group are more significant than 0.05. Moreover, all *p*-values used chi-square tests for three SNPs are less than 0.001 (Table 2).

### Analysis of Haplotypes Based on Six SNPs

Through linkage analysis of six SNPs such as IVS6-30G > T, GTT^527^/GTC^527^, A^669^TG/G^669^TG, C^987^GG/G^987^GG, TAA^1118^/TAC^1118^, and C^1178^/A^1178^, we found that Chinese MJD patients only shared 7 haplotypes, while healthy people shared 21 haplotypes (Table 3). Among them, T-T-A-G-C-C only appears in the MJD patients. The remaining 6 haplotypes were confirmed statistically significant by chi-square test, and all the *p*-values were less than 0.001.

**Table 3:** Overall linkage disequilibrium analysis of Chinese families for intragenic haplotypes based on 6 SNPs.

	Frequency in			
Haplotype^a^	Control subjects	Subjects with MJD	δ^b^	*P*-value
T-T-A-C-A-C	0.283	0.482	0.278	<0.001
T-T-A-C-C-A	0.013	0	…	…
T-T-A-G-A-C	0.119	0.382	0.298	<0.001
T-T-A-G-C-C	0.000	0.045	0.045	…
T-T-A-G-C-A	0.003	0	…	…
T-T-G-G-A-C	0.013	0.009	0.003	<0.001
T-T-G-G-C-A	0.013	0	…	…
T-T-G-C-A-C	0.006	0.018	0.012	<0.001
T-C-G-G-C-A	0.019	0	…	…
G-C-G-G-C-A	0.321	0	…	…
G-C-G-G-A-C	0.006	0	…	…
G-C-G-C-C-A	0.035	0	…	…
G-C-G-C-A-C	0.013	0	…	…
G-C-A-G-C-A	0.028	0	…	…
G-C-A-G-A-C	0.009	0.009	0	<0.001
G-T-A-G-A-C	0.016	0.055	0.040	<0.001
G-T-A-G-C-A	0.003	0	…	…
G-T-A-C-A-C	0.009	0	…	…
G-T-A-C-C-A	0.009	0	…	…
G-T-G-G-A-C	0.003	0	…	…
G-T-G-G-C-A	0.066	0	…	…
G-T-G-C-C-A	0.013	0	…	…


*^*a*^Haplotype based on IVS6-30G > T, GTT^*527*^/GTC^*527*^, A^*669*^TG/G^*669*^TG, C^*987*^GG/G^*987*^GG, TAA^*1118*^/TAC^*1118*^, C^*1178*^/A^*1178*^*.

*^b^The approximate risk of population attribution.*

### Age Estimation

**FIGURE 2:**
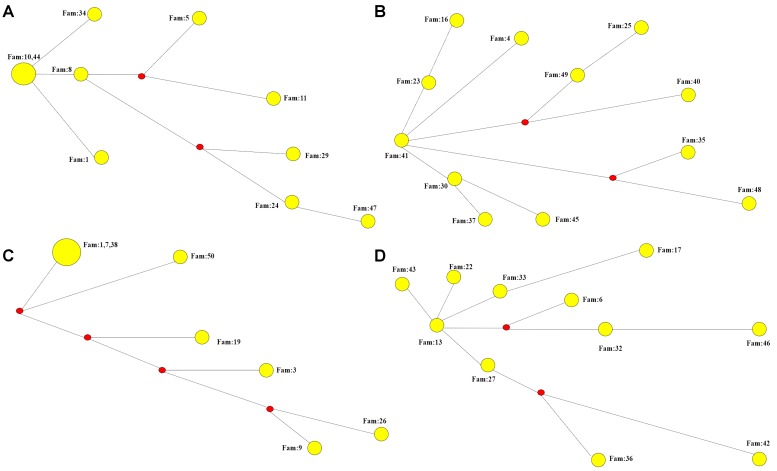
Phylogenetic networks of the four major haplotypes based on 7 microsatellite loci. **(A)** Phylogenetic network of haplotype A. **(B)** Phylogenetic network of haplotype B. **(C)** Phylogenetic network of haplotype D. **(D)** Phylogenetic network of haplotype G.

We performed STR analysis and age estimation on four major haplotypes A, B, D, and G based on 20 SNPs, and inferred the Phylogenetic networks of the four haplotypes (Figure 2). Surprisingly, MJD haplotypes A and D seemed to be present in the Chinese population as remote as 16,335 ± 1,966 and 11,837 ± 1,871 years, respectively; whereas introduction of haplotypes B and G probably occurred simultaneously, a few million years later, 9,272 ± 1,352 and 9,254 ± 1,411 years ago, respectively (Table 4).

**Table 4:** Haplotypes and age estimation with 7 STR flanking the (CAG)n at the *ATXN3*, from families sharing the four most common SNP-based haplotypes.

SNP-based haplotype	Family number	(TAT_223)n	(GT_199)n	(ATA_194)n	(AC_21)n	(AAAC)n	(GT)n	(AC_190)n	Age^a^
Haplotype A:TTGATCAAGC-(CAG)_exp_-CACCCAGCGC	Fam 10	10	20	10	13	7	16	15	16,335 ± 1,966
	Fam 44	10	20	10	13	7	16	15	
	Fam 34	11	20	10	13	7	15	15	
	Fam 1	10	20	10	13	7	20	18	
	Fam 8	10	22	10	13	7	16	15	
	Fam 5	10	26	10	13	7	16	15	
	Fam 11	10	24	10	12	7	14	18	
	Fam 24	14	22	10	12	8	20	15	
	Fam 29	16	22	10	13	7	20	15	
	Fam 47	16	24	10	14	5	20	16	
Haplotype B:TTGATCGAGC-(CAG)_exp_-CACCCAGCGC	Fam 41	10	22	10	12	7	20	16	9,272 ± 1,352
	Fam 23	10	22	10	13	7	20	16	
	Fam 16	10	21	11	13	7	20	16	
	Fam 4	11	24	10	12	7	20	15	
	Fam 49	10	22	10	12	7	16	16	
	Fam 25	10	22	10	14	7	16	16	
	Fam 40	10	22	9	12	7	17	19	
	Fam 30	10	22	9	12	7	20	16	
	Fam 37	10	22	9	12	8	20	16	
	Fam 45	10	20	9	12	7	20	16	
	Fam 35	15	22	8	12	7	20	16	
	Fam 48	16	22	9	12	6	20	15	
Haplotype D:TTGATCAAGC-(CAG)_exp_-GACCCAGCGC	Fam 1	10	20	10	13	7	16	15	11,837 ± 1,871
	Fam 7	10	20	10	13	7	16	15	
	Fam 38	10	20	10	13	7	16	15	
	Fam 50	11	19	12	13	7	15	15	
	Fam 19	13	22	9	13	7	16	15	
	Fam 3	16	23	10	13	7	16	15	
	Fam 9	17	21	10	13	7	17	15	
	Fam 26	16	21	10	13	7	20	15	
Haplotype G:TTGATCGAGC-(CAG)_exp_-GACCCAGCGC	Fam 13	10	22	9	12	7	20	16	9,254 ± 1,411
	Fam 43	10	22	8	12	7	20	16	
	Fam 22	10	22	9	12	7	20	15	
	Fam 33	10	22	9	15	7 (5)	19	16	
	Fam 17	10	22	9	14	8	16	16	
	Fam 6	12	22	10	12	7	20	16	
	Fam 32	16	22	9	12	7	20	16	
	Fam 46	16	21	10	12	7	20	15	
	Fam 27	10	22	9	12	5	20	16	
	Fam 36	10	22	10	12	5	20	15	
	Fam 42	11	22	11	12	5	20	20	


*^*a*^Age is the estimated age of occurrence for each haplotype based on 20 SNP.*

## Discussion

For the selection of participants, we require that the proband must have more than one children or proband’s family must bear more than two generations. The stringency of the selection criteria for the participants made only 5 of the families inferred as haplotypes by software, suggesting the vast majority of families inferred haplotypes through the pedigree structure to make our results more accurate and unquestionable.

The rs56268847 found in Asian SCA3/MJD patients was not statistically significant in the previous report (Martins et al., 2012), most probably due to the small sample size and different national backgrounds. Pathogenic chromosomes of 28 probands in 50 families carry G allele with a frequency of 0.56. The *p*-value of the Hardy-Weinberg equilibrium is <0.05 in the SCA3/MJD group, indicating that this allele does not reach a genetic equilibrium. However, the difference between the control group confirmed to achieve the genetic balance after the same test and Chi-square test, and the SCA3/MJD group is statistically significant. Thus, it is reasonable to speculate that the difference might be primarily caused by the disease. Furthermore, since the G allele at rs56268847 has been observed to segregate with the expanded allele in more than half of our Chinese MJD families, this suggests that a point mutation at this SNP must have occurred early in the introduction of MJD in China or, even, in other Asian countries.

Additionally, for the first time we detected base variations (new base addition) in three SNPs rs16999141, rs10467857, and rs77086230 in the Chinese SCA3/MJD patients compared to the previously reported two ancestral lineages. Both ancestral lineages are reported as T on rs16999141, and we detected T/C. While G is reported in the ancestral lineage SNP rs10467857, we detected G/C instead of G only. Similarly, C/T is found in the Chinese SCA3/MJD patients other than C only as reported in the ancestral lineage SNP rs77086230. As for the significance in our new discovery of the new base variation in 3 sites, although so far only one family were examined for each SNP, two test methods have confirmed the significant difference. To be more conclusive to say that the new base variations occurred relatively recently, tests of more families are required.

Previous study showed that the A-G-A haplotype for the SNPs rs1048755, rs12895357, and rs7158733 in the 249 of families, exist in MJD patient (Gaspar et al., 2001), while they were not statistically significant most probably due to the different backgrounds. More convincingly, the *p*-value obtained by Fisher’s exact test is less than 0.001. Thus, we believe the significant difference in the frequency of A-G-A haplotype between the SCA3/MJD and the control group.

The first studies on the epidemiology of MJD were based on families described before the gene ATXN3 was known (Sequeiros and Coutinho, 1993). Later, two major MJD haplotypes have been identified (Gaspar et al., 2001; Martins et al., 2007); here, in addition to the worldwide spread Joseph lineage, we found three other major haplotypes that differ from Joseph at (1) rs56268847 (lineage B, 12 families), (2) rs12895357 (lineage D, 8 families), and (3) both rs56268847 and rs12895357 (lineage G, 11 families). Interestingly, lineage B has been previously described among Australian aborigines, probably introduced in this population via Asia (Martins et al., 2012). Independent mutational origins do not necessarily underlie these four MJD SNP-haplotypes since recurrent mutations on the 2 SNPs may have occurred: backgrounds B and D may have evolved from the Joseph lineage by recurrence at rs56268847 and rs12895357, respectively; recombination could be a possible explanation for the origin of SNP backgrounds G, although the short distance between the two SNPs and the deleterious (CAG)n makes it unlikely. Other MJD backgrounds, phylogenetically more distant from the Joseph lineage, were found in single MJD families; de novo expansions may be on the origin of some of them, but a larger cohort should confirm that their low frequency is explained by a recent event or genetic drift.

This discovery is of importance to clarify the prevalence of SCA3/MJD and could become indispensable evidence that supports the founder effect in this disease. More importantly, this finding could help decipher the genetic basis of the SCA3/MJD by further study on the haplotypes.

## Informed Consent

Informed consent was obtained from all individual participants included in the study.

## Author Contributions

TL completed the collection of samples, analysis of data, and writing of the manuscript. SM, JS, ZH, KX, BT, and HJ completed the design of the experiments. YP, PW, XH, ZC, CW, and ZT assisted in the collection of samples. RQ and CC contributed to the analysis of the data.

## Conflict of Interest Statement

The authors declare that the research was conducted in the absence of any commercial or financial relationships that could be construed as a potential conflict of interest.

## Footnotes

1 http://stephenslab.uchicago.edu/phase/download.html
