# Supplementary material for: Is the High Frequency of Machado-Joseph Disease in China Due to New Mutational Origins?
Source: Front Genet. 2019 Feb 20;9:740. doi: 10.3389/fgene.2018.00740 (PMC6391318; doi:10.3389/fgene.2018.00740)
Supplement: Supplementary file 1 [file Table_1.DOCX]

**Table S1. The Distance from (CAG)n and Primers of Single Nucleotide Polymorphisms and Microsatellite Repeats.**

| Locus | Distance from  the (CAG)n (bp) | Primers |
| --- | --- | --- |
| (TAT_223)n | 223 315 | F: CCACAAGTGGTGCTAGCCTTTG  R: TCAGTGAGCTGAGATCGTGC |
| (GT_199)n | 198874 | F: CCTCAGGTCCCATAGGTTGGAATGTAA  R: GCCTCAACCTCCCAAGCTCAAG |
| (ATA_194)n | 194510 | F: CTTGTTTCACCTATCCCACCTC  R: GAAGCACATCCAAGACATCACATC |
| rs12590497 | 12352 | F: CAGTGTTCTGTGCTGCCTTT  R: TGCAGGCCTCATTTTTACCT |
| rs16999141 | 12306 | F: CAGTGTTCTGTGCTGCCTTT  R: TGCAGGCCTCATTTTTACCT |
| rs10146519 | 11616 | F: CCATCATTTGCTTCTAACACTC  R: AACTTAGTTGGATTCAAATTGC |
| rs1048755 | 11505 | F: TACTAGAGCTTATTTGCCAG  R: CAGAGCCCTCTGCAAATCCT |
| rs12586535 | 1249 | F: CAATTATTGGCCTTTCTGAACCA  R: CTGTGCTGTTACATAGTAAGCATTCAC |
| rs12586471 | 1125 | F: CAATTATTGGCCTTTCTGAACCA  R: CTGTGCTGTTACATAGTAAGCATTCAC |
| rs56268847 | 888 | F: GGTGTTCAGTGAATGCTTACTATG  R: GCAAATGAGTGTTGGTTTATAGACCC |
| rs10467858 | 505 | F: TTTGTTTGTTGTTTTTTTGAGAC  R: AAAAGAATGCAAGAGCAGTTAGT |
| rs10467857 | 463 | F: TTTGTTTGTTGTTTTTTTGAGAC  R: AAAAGAATGCAAGAGCAGTTAGT |
| rs10467856 | 452 | F: TTTGTTTGTTGTTTTTTTGAGAC  R: AAAAGAATGCAAGAGCAGTTAGT |
| (CAG)n |  |  |
| rs12895357 | 1 | F: CCAGTGACTACTTTGATTCGTGA  R: TTCTTTTGGTAACTGCTCCTTAA |
| rs7158733 | 132 | F: CCACCAGTTCAGGAGCACTT  R: GCTCCTTAATCCAGGGAAATTTAG |
| rs3092822 | 192 | F: CCACCAGTTCAGGAGCACTT  R: GCTCCTTAATCCAGGGAAATTTAG |
| rs77086230 | 1342 | F: GATCCAGCAGTCCCAATCATGTA  R: CTTCAGGTATCTGCCAACCTCG |
| rs79316375 | 1964 | F: GAGGCAGGAGAATCACTTGA  R: TCAATTGTTTGGATATACCACAG |
| rs8004149 | 2440 | F: TTGTATAAAACATGAGCTGGGCT  R: CTGGGAAAGGGGAGAAGTGT |
| rs111735934 | 2472 | F: TTGTATAAAACATGAGCTGGGCT  R: CTGGGAAAGGGGAGAAGTGT |
| rs181752420 | 2576 | F: TTGTATAAAACATGAGCTGGGCT  R: CTGGGAAAGGGGAGAAGTGT |
| rs7142326 | 2614 | F: TTGTATAAAACATGAGCTGGGCT  R: CTGGGAAAGGGGAGAAGTGT |
| rs74071847 | 2742 | F: TTGTATAAAACATGAGCTGGGCT  R: CTGGGAAAGGGGAGAAGTGT |
| (AC_21)n | 20 817 | F: GATGTGATGTCTTGGATGTGCTTC  R: CTTGTTTCACCTATCCCACCTC |
| (AAAC)n | 122626 | F: CAGATGGGATAGGCCACAGT  R: AGTGGAGGCTTCAACCTGTT |
| (GT)n | 189 857 | F: GAGGGGACCTGGCCTACTAC  R: GCACTGCAAACCAGTAGCAC |
| (AC_190)n | 190368 | F: CTGGGAGGAGGAGGGTACAA  R: AACCCTGACTCAACTCTCGG |

F: represents forward primer; R: represents reverse primer
